# Paraneoplastic Syndrome in Adrenocortical Carcinoma: The Unexpected Mime

**DOI:** 10.7759/cureus.63534

**Published:** 2024-06-30

**Authors:** Corina Nava Suarez, Janna Prater, Jane Mayrin, Galyna Vorokhib, Minimo Corrado

**Affiliations:** 1 Endocrinology, Jefferson Einstein Philadelphia Hospital, Philadelphia, USA; 2 Pathology, Jefferson Einstein Philadelphia Hospital, Philadelphia, USA

**Keywords:** cushing's syndrome, pulmonary embolism (pe), thrombosis, endogenous hypercortisolism, adrenocortical carcinoma (acc)

## Abstract

Adrenocortical carcinoma (ACC) is a malignancy of the adrenal cortex with a high morbidity and mortality. More than half of the cases are functional tumors. As different hormones can be co-secreted above physiologic levels, it causes a very broad variety of symptoms and makes differentiating from more common entities hard. Here we present a case of a patient with a newly diagnosed ACC who initially presented with acute pulmonary embolism and recurrent deep vein thromboses (DVT) in the setting of hypercortisolism. Imaging showed a left adrenal mass invading adjacent structures including a nonocclusive thrombus in the left renal vein. Intravenous anticoagulation and thrombectomy were initially performed, followed by removal of the tumor and adjacent metastatic disease. Pathology confirmed ACC. The patient underwent left adrenalectomy, left nephrectomy, splenectomy, distal pancreatectomy, and caval thrombectomy with inferior vena cava (IVC) filter placement. Intravenous anticoagulation and glucocorticoid replacement were also administered as part of the treatment plan. Unfortunately, the patient had multiple episodes of bleeding and thrombosis and was eventually discharged to hospice care. DVT in the setting of ACC can be caused by increased hypercoagulability from hypercortisolism, direct venous thrombosis, or vascular invasion. Thrombosis, especially in the inferior vena cava, has been associated with poor prognosis and survival rates. Clinicians should be aware of this rare complication given its immediate therapeutic repercussions and prognostic value.

## Introduction

Adrenocortical carcinoma (ACC) is an infrequent neoplasm arising from the adrenal gland cortex that is associated with poor prognosis [1]. More than half of ACC cases are functional [2] and, consequently, manifest a broad variety of symptoms that can make it challenging to differentiate from more frequent diagnoses. Hypercortisolism is the most frequent hormonal excess; aldosterone, catecholamines, and androgens can also be secreted above physiologic levels [2,3], and co-secretion of more than two adrenal hormones should raise suspicious of a possible ACC [2]. Thrombosis has been reported in a few case series in the literature, but it is an infrequent complication (2.9%) and has been associated with a grim prognosis [1,3-5].

This article was previously presented as a meeting ePoster at the 2024 American Association of Clinical Endocrinology (AACE) Annual Meeting on Thursday, May 9, 2024.

## Case presentation

A 68-year-old female with long-standing type 2 diabetes mellitus, prior pulmonary embolism (PE) in the setting of COVID-19 infection, asthma, and coronary artery disease was sent to the emergency department (ED) from her endocrinologist’s office after she complained of fatigue, unsteadiness, dizziness, and diaphoresis during a regular visit. She also endorsed a 20-pound weight loss over six months. Diabetes became difficult to control in the three to four months before the visit requiring higher doses of insulin. Point-of-care finger stick blood glucose was 547 mg/dL. Once in the ED, the patient was found to be tachycardic, and her physical exam was significant for a round face and central obesity raising concern for excess cortisol secretion.

**Figure 1:**
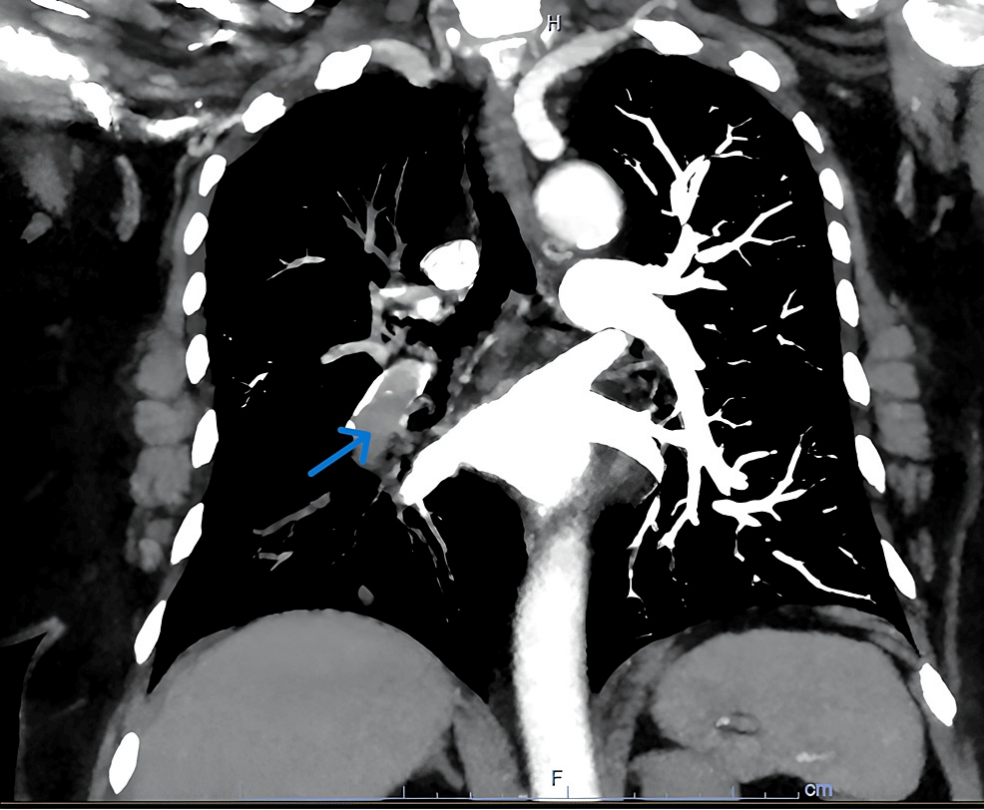
CT pulmonary embolism protocol Arrow showing a filling defect in the right main/interlobar pulmonary arteries representing a pulmonary embolism

**Figure 2:**
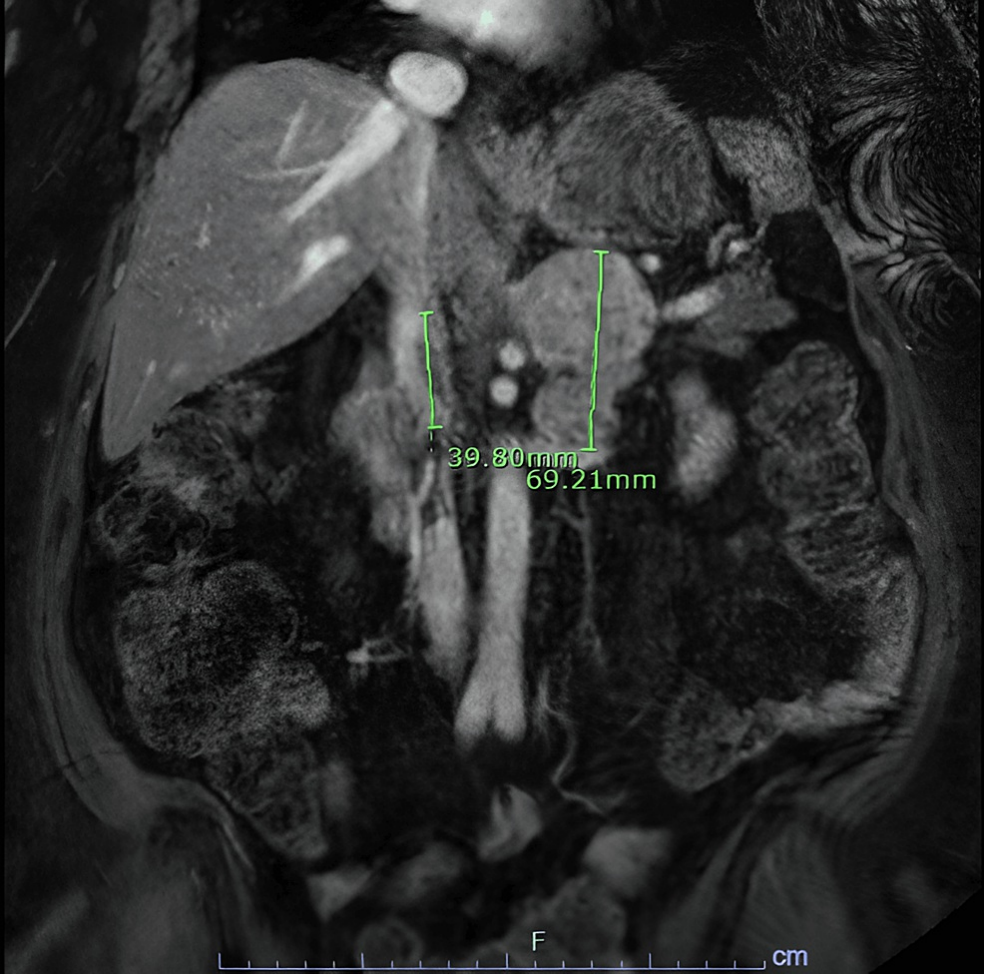
Adrenal MRI with contrast (coronal view) The left mark shows a nonocclusive thrombus in the IVC, and the right mark shows a 69.21 mm bilobated adrenal mass with tumor invasion to the left renal vein.

As part of her workup, a CT angiography of the chest demonstrated an acute right main pulmonary embolism (Figure 1) with right ventricular strain, indeterminate 12 mm pulmonary nodules, and left external iliac vein thrombosis. A CT abdomen and pelvis with IV contrast showed a solid bilobed enlargement of the left adrenal gland and an additional infra-adrenal mass abutting the left renal vein with features suggestive of a thrombus. A hormonal workup suggested hypercortisolism of adrenal origin with a significantly elevated cortisol after the administration of 1 mg dexamethasone and a suppressed adrenocorticotropic hormone (ACTH). Her dehydroepiandrosterone sulfate (DHEA-S) was also high, suggestive of hyperandrogenism and co-secretion of adrenal hormones. Serum normetanephrine was marginally elevated and attributed to her acute illness (Table 1). Adrenal MRI with and without contrast confirmed the presence of a 6.9 x 5.6 x 5.2 cm bilobed adrenal mass extending inferiorly in the left retroperitoneum. The mass extended into the left renal and splenic veins, the greater curvature of the stomach, and a nonocclusive thrombus in the left renal vein and inferior vena cava were present (Figure 2).

**Table 1:** Biochemical evaluation The initial hormonal workup of the patient showed hypercortisolism and hyperandrogenism from an adrenal origin, uncontrolled hyperglycemia, and mildly elevated serum normetanephrine. ACTH: adrenocorticotropic hormone; DHEA-S: dehydroepiandrosterone sulfate; HbA1c: glycated hemoglobin; PRA: plasma renin activity

Test name	Value	Reference level
AM cortisol	25	3.7-19.4 mcg/dL
AM cortisol after 1 mg dexamethasone	19.8	3.7-19.4 mcg/dL
ACTH	<5	6-50 pg/mL
DHEA-S	328	12-133 mcg/dL
Free testosterone	25.6	0.1-6.4 pg/mL
Total testosterone	42	2-45 ng/dL
Aldosterone	2	3-16 ng/dL
Renin PRA	2.98	0.25-5.82 ng/mL/hr
Free metanephrine	32	<57 pg/mL
Free Normetanephrine	284	<148 pg/mL
Total, free metanephrine and normetanephrine	316	<205 pg/mL
HbA1c	>14%	<5.7%

**Figure 3:**
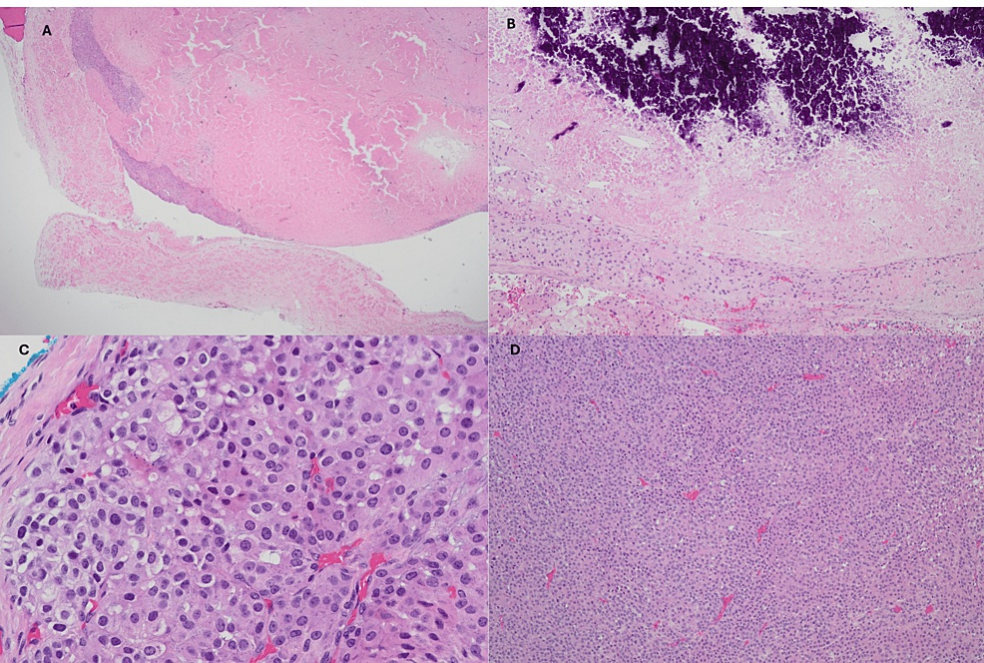
Pathology slides Pathology slides showing (A: H&E 2X) solid oncocytic ACC with vascular invasion, necrosis, and (B: H&E 10X) calcifications. It had a (C: H&E 40X) high mitotic count, with <25% clear cells and (D: H&E 10X) >30% solid component with atypical mitosis.

IV heparin was started as a bridge to a right pulmonary arterial thrombectomy. The patient also underwent left adrenalectomy, left radical nephrectomy, splenectomy, distal pancreatectomy, and caval thrombectomy with IVC filter placement. After the procedure, IV dexamethasone was initiated for the management of secondary adrenal insufficiency. Pathology confirmed oncocytic adrenal cortical carcinoma with high-grade mitoses, extensive necrosis, and calcifications (Figure 3) and a Ki-67 labeling index of 30%.

The postsurgical course was complicated by the formation of a right abdominal wall and liver hematomas requiring exploratory laparotomy and hematoma evacuation. She had a slow recovery during the next one to two months but was very debilitated and frail with associated anorexia and limited mobility. Eventually, the patient was able to be discharged to a skilled nursing facility and then needed to be readmitted to our institution after nine days with severe swelling of the left lower extremity, hypotension, and altered mental status. She was found to have increased thrombus burden in the left external iliac vein with extension into the popliteal vein. Ultimately, the patient was discharged to her home with hospice care and lost follow-up to our institution.

## Discussion

ACC is a neoplasm that arises from the adrenal cortex; it has an estimated incidence of about 2/1,000,000 people/year and a five-year survival of 15-44% [1]. More than 50% of ACCs are functional masses, with cortisol being the most frequent hormone overproduced, and about 30% of patients show clinical manifestations from hypercortisolism [2,3]. The excess of other adrenal hormones such as aldosterone, catecholamines, and androgens has also been described [2]. The presence of more than one hormonal excess should raise suspicion of a potential adrenal malignancy [6]. Diagnosis can be challenging as symptomatology can be vague and vary if there is an associated hormonal overproduction; however, rapidly progressive Cushing’s syndrome with associated virilization should always raise concerns for ACC [2,6]. In some asymptomatic patients, the diagnosis occurs as an incidental finding during an imaging study performed for a different purpose [6-8], as in our patient’s case. Heterogeneity, irregular borders, calcifications, necrosis, and a lipid-poor mass with a high attenuation value (higher than 10 Hounsfield units (UH)) on an unenhanced CT or a mass larger than 4 cm are all features that could be suggestive of adrenal malignancy on CT imaging [6,8,9]. Dedicated adrenal MRI does not offer a better characterization of the mass; nonetheless, it can better assess invasion into the inferior vena cava and should be used for patients on whom exposure to radiation is undesired [6]. More rarely, patients can initially present with thrombosis (less than 3% of cases [3]), with even more sporadic cases of pulmonary and intracardiac embolism in the literature [5].

ACC can be histologically classified as conventional, oncocytic, myxoid, and sarcomatoid, with the first one being the most common and only 56 cases of oncocytic ACC reported in the literature by 2021 [10]. The Weiss score is a validated tool for microscopic diagnosis of ACC. This includes the presence of a high mitotic count and/or nuclear grade, capsular, sinusoidal and/or vascular invasion, atypical mitosis, presence of necrosis, diffuse architecture, and <25% clear cells. A high mitotic count (>20/50 HPF) and proliferation index and Ki67 immunomarker >10% are both used as prognostic markers and high recurrence rate [7,10].

The development of venous thromboembolism in ACC can have multiple causes. Hypercortisolism appears to increase clotting factors VIII and fibrinogen and impairs fibrinolysis [11,12]. Direct venous thrombosis, or vascular invasion leading to direct endothelial injury, is another potential mechanism [5]. There are also case series reporting case-related thrombotic microangiopathy in ACC [4]. Our patient was also very frail and immobile, which could have added venous stasis as a third risk factor. The presence of thrombosis, especially in the inferior vena cava, has been associated with poor prognosis and survival rates [3-5]. Complete surgical resection is the only possible curative option for ACC [1,2,7]. Unfortunately, the majority of patients present with distant metastases at the time of diagnosis, and adjuvant therapies such as mitotane and systemic chemotherapy are sometimes used in these circumstances; however, prognosis remains poor in patients with advanced disease [4,5,10].

## Conclusions

ACC is an infrequent malignancy of the adrenal cortex with a broad symptomatology as tumors can be both functioning and nonfunctioning making it difficult to differentiate from more common entities in most instances. It carries a high morbidity and mortality especially when complicated by thrombotic events. Clinicians should be aware of this entity and familiarize themselves with the possible clinical presentation as a high index of suspicion is usually required to diagnose ACC and to improve outcomes. The treatment for ACC is limited especially when diagnosed at an advanced stage. Further research is needed to expand new therapeutic options that could improve prognosis.
